# Frequent retrotransposition of endogenous genes in ERCC2-deficient cells derived from a patient with xeroderma pigmentosum

**DOI:** 10.1186/s13287-019-1381-z

**Published:** 2019-08-27

**Authors:** Saki Aoto, Saki Katagiri, Yi Wang, Alistair T. Pagnamenta, Rie Sakamoto-Abutani, Masashi Toyoda, Akihiro Umezawa, Kohji Okamura

**Affiliations:** 1 Medical Genome Center, National Center for Child Health and Development Research Institute, Setagaya, Tokyo, Japan; 2 Department of Biology, Faculty of Science, Ochanomizu University, Bunkyo, Tokyo, Japan; 3 Ministry of Education Key Laboratory of Contemporary Anthropology, Department of Anthropology and Human Genetics, School of Life Sciences, Fudan University, Shanghai, China; 4 Human Phenome Institute, Fudan University, Shanghai, China; 5 NIHR Oxford BRC, Wellcome Centre for Human Genetics, University of Oxford, Oxford, UK; 6 Department of Reproductive Biology, National Center for Child Health and Development Research Institute, Setagaya, Tokyo, Japan; 7 Research team for Geriatric Medicine, Tokyo Metropolitan Institute of Gerontology, Setagaya, Tokyo, Japan; 8 Department of Systems BioMedicine, National Center for Child Health and Development Research Institute, Tokyo, Japan; 9 Center for Regenerative Medicine, National Center for Child Health and Development Research Institute, 2-10-1 Okura, Setagaya, Tokyo, 157-8535 Japan; 10 Present address: Division of Embryology, National Institute for Basic Biology, Okazaki, Aichi Japan

**Keywords:** Retrotransposition, ERCC2, XPA, Xeroderma pigmentosum, DNA repair, Somatic mutation, iPSC

## Abstract

**Background:**

Retrotransposition of protein-coding genes is thought to occur due to the existence of numerous processed pseudogenes in both animals and plants. Unlike retrotransposons including *Alu* and LINE-1, direct evidence of such retrotransposition events has not been reported to date. Even if such an event occurs in a somatic cell, it is almost impossible to detect it using bulk of cells as a sample. Single-cell analyses or other techniques are needed.

**Methods:**

In order to examine genetic stability of stem cells, we have established induced pluripotent stem cell (iPSC) lines from several patients with DNA repair-deficiency disorders, such as ataxia telangiectasia and xeroderma pigmentosum, along with healthy controls. Performing whole-exome sequencing analyses of these parental and iPSC lines, we compiled somatic mutations accumulated by the deficiency of DNA repair mechanisms. Whereas most somatic mutations cannot be detected in bulk, cell reprogramming enabled us to observe all the somatic mutations which had occurred in the cell line. Patterns of somatic mutations should be distinctive depending on which DNA repair gene is impaired.

**Results:**

The comparison revealed that deficiency of ATM and XPA preferentially gives rise to indels and single-nucleotide substitutions, respectively. On the other hand, deficiency of ERCC2 caused not only single-nucleotide mutations but also many retrotranspositions of endogenous genes, which were readily identified by examining removal of introns in whole-exome sequencing. Although the number was limited, those events were also detected in healthy control samples.

**Conclusions:**

The present study exploits clonality of iPSCs to unveil somatic mutation sets that are usually hidden in bulk cell analysis. Whole-exome sequencing analysis facilitated the detection of retrotransposition mutations. The results suggest that retrotranspositions of human endogenous genes are more frequent than expected in somatic cells and that ERCC2 plays a defensive role against transposition of endogenous and exogenous DNA fragments.

## Background

Xeroderma pigentosum (XP) is a rare hereditary disease characterized by high sensitivity to ultraviolet (UV) rays and development of skin tumors. The condition arises as a result of defective DNA repair mechanisms and is inherited in an autosomal recessive manner. Causative genes have been linked to several steps of the nucleotide excision repair pathway. By performing genetic complementation analyses, XP has been subdivided into 8 groups. Each complementation group has been associated with a specific causative gene as well as distinctive patient prognoses. Fibroblast cell lines derived from XP patients also exhibit different cytological phenotypes, such as unscheduled DNA synthesis and colony formation after UV exposure, depending on which group the samples belong to. In Japan, xeroderma pigmentosum complementation group A (XP-A), which involves the most severe cutaneous and neurological symptoms, is the most common form, followed by XP-V, XP-D, and XP-F [1]. XP-V patients exhibit mild phenotypes with almost normal DNA repair ability. The frequency of XP is more than 10 times higher in Japan than that in the USA or Europe. XP-C is the most common type in Western countries [2, 3] and XP-B has not been reported in the Japanese population. Whereas many Western XP-D patients exhibit neurological abnormalities, Japanese XP-D patients do not.

The XPA protein, which is responsible for XP-A, is a damage recognition protein that binds to DNA lesions. XPC, which is responsible for XP-C, works as an initiator of the nucleotide excision repair (NER) pathway by spotting UV-induced formation of dipyrimidine [4]. XPB and XPD, which are causative genes for XP-B and XP-D, respectively, are helicase enzymes of the transcription and repair complex TFIIH. They are thought to play a pivotal role in opening double-stranded DNA and removing lesions, and exhibit the opposite polarity of helicase. Mutations in the *XPB*, *XPD*, or *XPG* genes have been shown to be associated with Cockayne syndrome and trichothiodystrophy, which are characterized by premature aging [5–7]. Some of these patients share photosensitive symptoms of XP. Clinical and cytological phenotypes of the patients depend on which gene and which domain bears the causative mutation.

The eight complement groups show different DNA repair ability. For example, XP-A and XP-C patients suffer juvenile onset of skin cancers, while other XP patients tend to show onset in their 40s [1]. Each group is thought to bear characteristic mutational signatures depending on the nature of the causative mutation. In our previous study, in order to scrutinize DNA repair genes, we generated induced pluripotent stem cell (iPSC) lines from patients diagnosed with a DNA repair-deficiency disorder and compared the accumulation of somatic mutations between the parental fibroblasts and the iPSC lines to identify so-called passenger mutations, which were the results of the causative mutation. The comparisons revealed distinct features in mutational patterns between double-strand break repair and nucleotide excision repair deficiencies [8]. In ATM-deficient cells, while a large number of structural and short indel mutations had accumulated, the number of single-nucleotide mutations detected was negligible. In XPA-deficient cells, however, more than 10 times the number of single-nucleotide mutations were observed when compared with the number found in the ATM-deficient cells. Dipyrimidine-related mutations accounted for more than 80% of all the single-nucleotide mutations. Structural mutations were not detected, and the total number of indel mutations was less than that of ATM-deficient cells.

Disease models using human induced pluripotent stem cells (iPSCs) have recently been described as a promising tool for the investigation of disease mechanisms and identification of new drugs [9–13]. In the present study, we generated an iPSC line from fibroblasts derived from a Japanese patient diagnosed as suffering from XP-C and investigated the differences in genomic phenotypes, i.e., mutation types, among DNA repair-deficient cell lines. Whole-exome sequencing analysis showed that the patient should have been diagnosed with XP-D and suggested that XPD, also known as ERCC2, plays a role in suppressing retrotransposition of exogeneous and endogenous genes.

## Methods

### Human cells

The XP40OS fibroblast cell line was obtained from the JRCB Cell Bank, http://cellbank.nibiohn.go.jp/. It was originally derived from a skin specimen of a 30-year-old Japanese male diagnosed with xeroderma pigmentosum. Hypersensitivity to sunlight exposure was observed since his infancy. Neurological abnormalities, which are seen in some xeroderma pigmentosum patients, were not observed [14]. Its iPSC line was established using Sendai virus at the National Center of Child Health and Development [15, 16].

### Whole-exome sequencing

Genomic DNA samples were enriched with the SureSelect Human All Exon V4 + UTRs + lincRNA kit and the genome libraries were prepared according to the manufacturer’s instruction. These were sequenced on Illumina HiSeq 1000 with a 101-cycle paired-end mode. Reads were aligned to the GRCh38 and decoyJRGv1 sequences, which are available at Integrative Japanese Genome Variation [17], using the MEM algorithm of BWA 0.7.13. BAM files were prepared using SAMtools 1.3. PCR duplicate reads were removed using Picard 2.1.1. Local realignment, base recalibration, and variant call were performed using Genome Analysis Toolkit (GATK) 3.5.0. Multi-sample calling by GATK HaplotypeCaller was used to identify SNVs and indels among all the 13 samples with our in-house control samples. Somatic mutations were also identified in this way. Filtration of called variants was the same as our preceding study [18]. After following the GATK best practices, gene-based annotation was performed with ANNOVAR [19]. Only nonsynonymous and splicing-related variants were considered to narrow the search for causative mutations.

### Mutational signatures

For drawing the mutational signatures, only single-nucleotide substituted sites between an iPSC and its precursor cell samples were considered. Variants that did not pass the filtration process or that were not supported with 8 or more reads were eliminated. SNP genotyping was performed using HumanCytoSNP-12 v.1 DNA Analysis BeadChip Kit (Illumina) in order to find structural mutations, including deletion, copy-number gain, and copy-neutral loss of heterozygosity, in iPSCs. Single-nucleotide variants in a structural mutation were also eliminated for further analyses. Most of the remaining variants were found to be heterozygous. A few of homozygously detected variants, probably due to somatic mutation followed by gene conversion, were included. Sequencing and SNP-array data have been submitted to NCBI SRA and GEO under accession numbers SRP059858 and GSE55520, respectively.

### Detection of retrotransposed genes

To detect retrotransposed genes, we searched for candidate regions using the SAM-formatted data. Mapped paired reads with MAPQ ≥ 20, with ostensible template length ≥ 1000 bp, mapped to exonic regions with more than 1 read per exon, and spanning more than 2 discrete exons were listed as candidates of endogenous retrotransposed genes. Genomic coordinates of exonic regions were obtained from the UCSC Table Browser, https://genome.ucsc.edu/cgi-bin/hgTables. Retrotransposed genes that had been incorporated into the reference sequence were not detected in this way.

## Results

In our previous report, we failed to identify etiologic mutations in the *XPC* gene in XP40OS cells which had been classified as the complementation group C. Since xeroderma pigmentosum is an autosomal recessive disorder and its frequency in the Japanese population is one in 22,000 [2], we searched for rare variants or mutations resulting in a homozygous or compound heterozygous state. Homozygous variants were found in 10 genes and five of them are common in the Japanese population. Others seemed to be unrelated to xeroderma pigmentosum phenotypes. Compound heterozygous mutations were found in 12 genes. According to the 1000 Genomes Project [20], the Human Genetic Variation Database [21], and the Integrative Japanese Genome Variation Database [17], only two mutations in the *ERCC2* locus turned out to be less than 0.01 of allele frequencies. *ERCC2* was formerly known as *XPD*, whose defect can result in several severe disorders including xeroderma pigmentosum complementation group D.

**Fig. 1:**
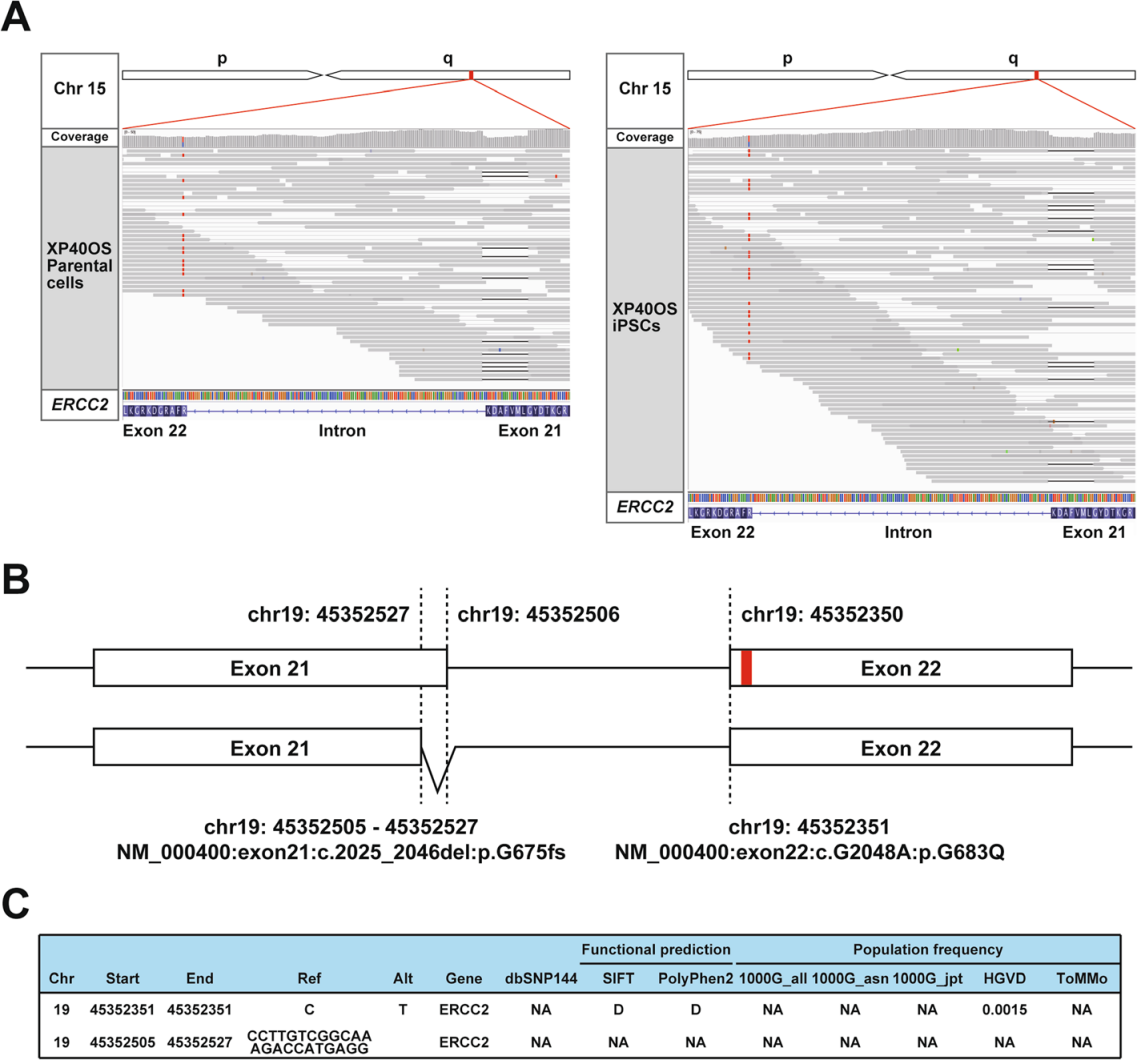
Compound heterozygous mutation identified at the *ERCC2* locus in XP40OS cells. **a** Integrated genome browser view. **b** Scheme of the exon 21 and 22 of the ERCC2 gene alleles with the compound heterozygous mutations. It consisted of a single-nucleotide substitution at 5′ end of an exon and a 23-bp deletion at 3′ end of its immediate upstream exon. Because the distance of the two was less than 200 bp that was strode by a number of single read pairs, the compound heterozygosity was readily revealed. **c** Mutation frequency and functional prediction of the mutation and deletion in the ERCC2 gene alleles with the ANNOVAR program

One of the two mutations was a single-nucleotide nonsynonymous substitution at GRCh38 position 45,352,351 (GRCh37 position 45,855,609) on chromosome 19. This nonsynonymous mutation rs758439420 (C to T) predicts a p.Arg683Gln change to the protein sequence (NP_000391.1) and had been found in several xeroderma pigmentosum patients. It is listed as a pathogenic variant in ClinVar (SCV000320713.1). Another mutation was a 23-bp deletion at GRCh38 position 45,352,505-45,352,527 (GRCh37 position 45,855,763-45,855,785) on chromosome 19 which disrupts the protein-coding sequence and its adjoining splice donor site (Fig. 1). An identical deletion was seen in the Genome Aggregation Database (gnomAD, v2.1.1) but with only one allele among 18,390 East Asian or 251,318 total alleles globally. In order to understand whether the two mutations form a compound heterozygous state in the *ERCC2* gene, haplotype phasing was carried out by visualizing read alignments using the Integrated Genomics Viewer (version 2.4.6). Because the two mutations are positioned close together, approximately 150 bp apart, on helicase domain VI, we readily found that each read-pair bears only one of the two variants, confirming compound heterozygosity (Fig. 1). As deleterious mutations were not found in the *XPC* locus, this case should be typed as complementation group D from the whole-exome analysis. The complementation analysis with the XPA-deficient and XPC-deficient cells revealed that XP40OS should be classified as group C [14]. Genetic complementation analyses of XP40OS were performed with only XPA-deficient and XPC-deficient cells, and this difference was observed between the two. If XPD-deficient cells had been employed, more precise results would have been obtained.

**Fig. 2:**
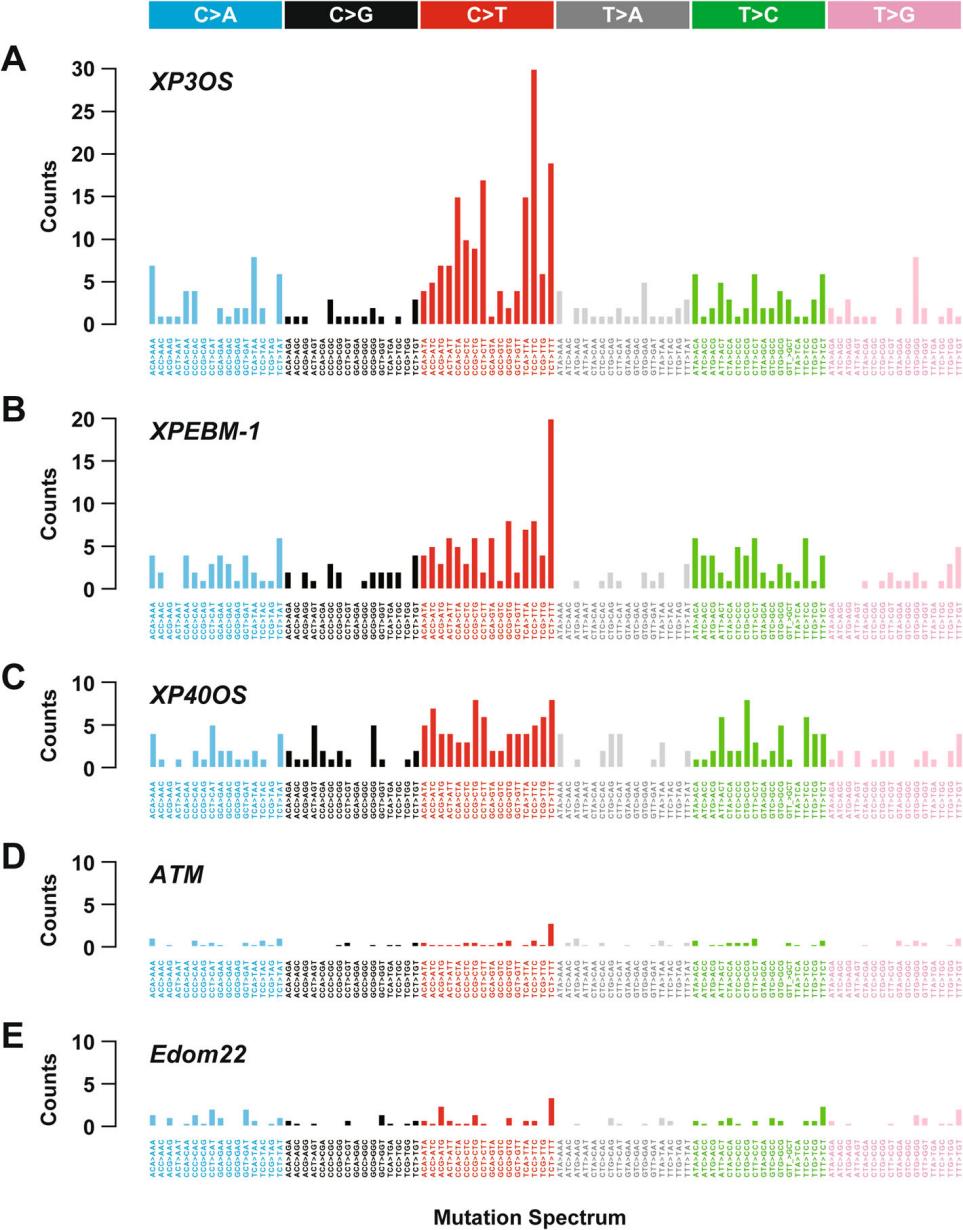
Mutational signatures of cell lines with or without DNA repair deficiency. There are 96 possible mutation types in total when trinucleotides in which the single-nucleotide mutation is situated in the center are considered. Vertical axes indicate the number of single-nucleotide mutations per cell line. Compared to cell lines deficient in repair of double-strand break and control cells, those deficient in nucleotide excision repair showed larger numbers of single-nucleotide mutation. Dipyrimidine spikes were only conspicuous in the *XPA*-deficient cells. **a** XPAiPS-O1 iPSCs and their parental XP3OS cells with the XPA mutations. **b** XP40OS iPSCs and their parental XP40OS cells with the ERCC2 mutations. **c** XPAiPS-E3 iPSCs and their parental XPEMB-1 cells with the XPA mutations. **d** AT-iPSCs (AT1OS-iPS_262, AT1OS-iPS_263, AT1OS-iPS_264, and AT1OS-iPS_024) and their parental AT1OS cells with the ATM mutations. **e** Edom-iPS-1, Edom-iPS-2, and Edom-iPS-3 iPSCs (Nishino et al. 2014) and their parental Edom22 cells from a healthy donor

In our previous studies, we established ATM-deficient and XPA-deficient iPSC lines and reported distinctive features of somatic mutations between the cell lines [8, 18]. The clonality of iPSC lines enabled us to detect mutations that had been buried in the bulk of cells. The precursor cells were derived from fibroblasts of ataxia telangiectasia and xeroderma pigmentosum complementation group A patients. Patients with these diseases have an increased risk of cancer due to deficiencies of DNA repair. The *ATM* and *XPA* genes are implicated in the recognition of double-strand breaks and nucleotide excision repair, respectively. In addition to these cell lines, we examined the mutational signatures of XP40OS and compared them to uncover deficiency type-specific features in the accumulation of somatic mutations (Fig. 2). For comparison, Edom22 parental cells and the respective iPSCs from a healthy woman were used. In addition, AT1OS, XP3OS, XP40OS, and XPEMB-1 were also considered as reciprocal controls because of the lack of the *ERCC2* mutation. Somatic retrotranspositions and other mutations were undetected in these controls, i.e., parental cells and their iPSCs. In the XPA-deficient cells, C-to-T mutations accumulated and were notably increased at dipyrimidine sites, which were observed as spikes in the mutational signatures. In XP40OS, the ERCC2-deficient cell line, C-to-T mutations were accumulated as in the XPA-deficient cell line, but the dipyrimidine spikes were not observed. The spikes most likely resulted from an inability of XPA to recognize UV-induced DNA lesions, such as thymidine dimer formation. Similar mutational features have been reported in urothelial tumor samples, in which some somatic mutations had been found in the *ERCC2* locus [22]. We identified structural variants in the cell lines by SNP-genotyping microarrays [8, 15, 18]. Single-nucleotide variants in these regions were eliminated from consideration.

In addition to nucleotide excision repair, DNA helicase ERCC2 may play a role in defense from retroviral infection. ERCC2- or ERCC3-deficient cell lines showed high efficiencies of transduction by human immunodeficiency virus or Moloney murine leukemia virus compared to XPA-deficient cells [23]. We did not observe substantial differences in efficiency in iPSC induction. In order to detect differences in retrotranscription and genome integration, we searched for retrotransposition of endogenous genes in each cell line. Like retrotransposons, such as LINE-1, any transcribed RNA molecules could be integrated into the genome by utilizing endogenous or viral reverse transcriptase activity [24]. Whole-exome sequencing is a technique for sequencing protein-coding regions in genomes. Compared to the source gene locus, a retrotransposed fragment would lack intronic sequences and map to the source region of the reference sequence. While most of the human mRNA molecules undergo splicing, there are some intronless transcripts. When an intronless gene is transcribed, reverse transcribed, and integrated, the retrotransposition is hard to be distinguished from segmental duplication. Hence, we limited targets to genes that bear introns and counted the number of such retrotransposition events in each cell line. Although whole-genome sequencing is preferable for such an analysis, whole-exome sequencing is also able to pick up such events and at an appreciably lower cost. Genomic fragments that had been subject to splicing were mapped to exonic sequences just as in RNA-seq mapping.

**Fig. 3:**
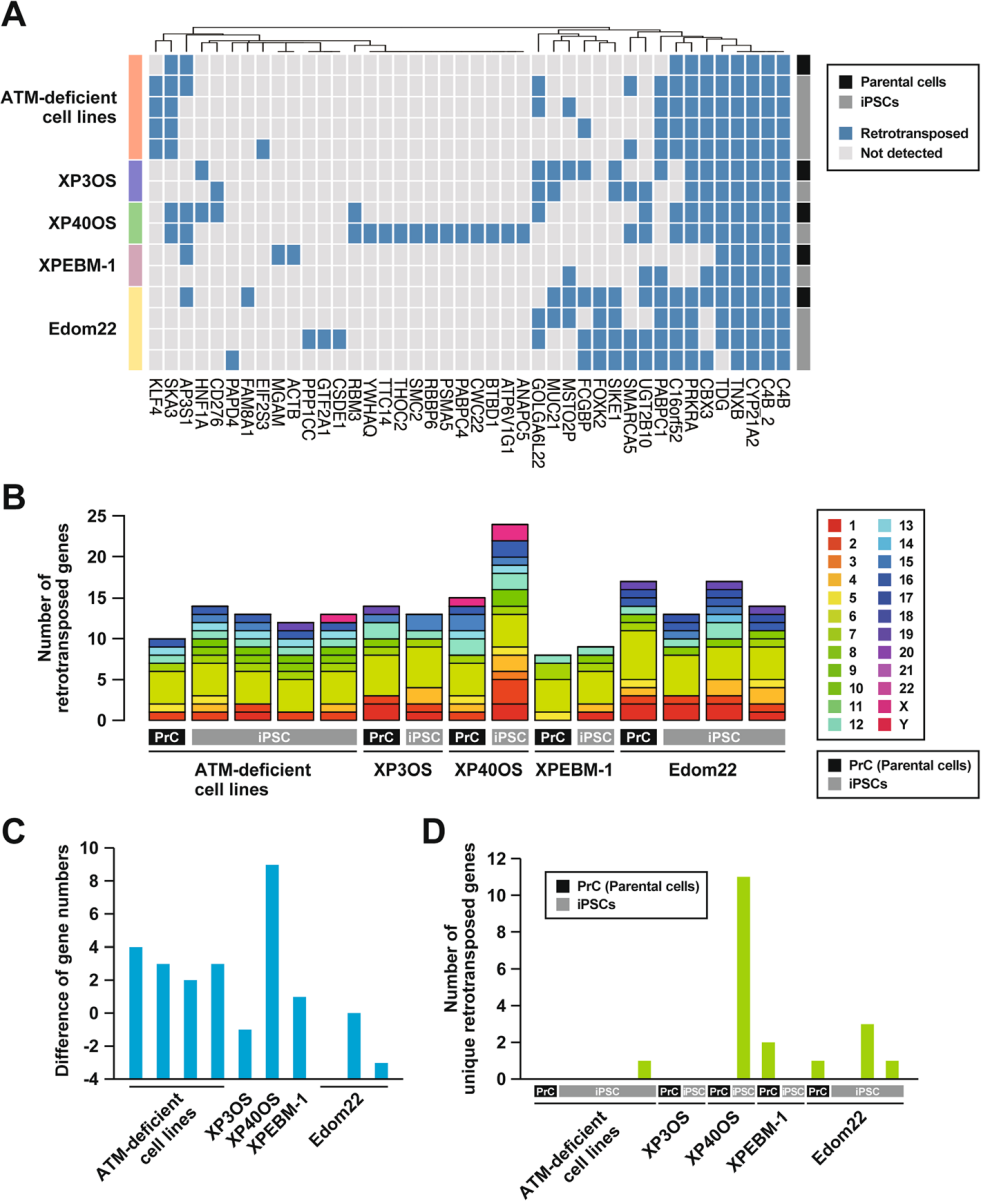
Retrotranspositions identified in each cell line. **a** Hierarchical clustering analysis of retrotransposed genes detected in the whole-exome sequencing. Each blue box indicates an existence of an intronless sequence, which is not assembled in the reference sequences. The two cell types, namely parental cells or iPSCs, are indicated by black and gray bars, respectively. **b** Numbers of retrotranspositions colored by their source, but not integrated, chromosomal positions. XP40OS iPSCs showed the largest number of retrotranspositions among all the samples in the present study. **c** Number of retrotranspositions in iPSCs subtracted by that in their parental cells. **d** Number of retrotransposed genes that were exclusively identified in a sole cell line

In total, 42 genes were first listed as candidates from which intronic sequences had been removed before genomic integration in the samples (Fig. 3). However, many of them seemed to be genomic relics. If their intronless forms were not assembled in the reference sequence, such a relic is detected as a candidate by this method. Our program detected retrotranspositions in all the samples of as for the *C4B*, *C4B_2*, *CYP21A2*, and *TNXB* genes. We noticed that their paralogous sequences are situated in their same vicinity in the reference sequence. It is therefore likely that the intronless sequences were not assembled in the reference sequence. The retrotransposed sequences of the *TDG* and *CBX3* genes had been compiled in the UCSC list of retroposed genes. That of *SKA3* was frequently found in the 1000 Genomes Project data, except in the Yoruba population [25, 26]. The *C16orf52*, *PABPC1*, *AP3S1*, and *PRKRA* genes were registered in retrogeneDB [27]. These retrotranspositions were detected in more than two cell lines in our study, suggesting high reliability of this exome-based method. Finally, 11 retrotransposed genes were exclusively found in the iPSC line of XP40OS (Fig. 3). Although unique retrotransposed genes were also detected in other cells, the number of them in the iPSC line of XP40OS was extremely high.

## Discussion

Because the first genetic complementation analysis of XP40OS cells had suggested that the patient might be suffering from XP-C, we searched for causable mutations in the *XPC* locus [14]. Only two heterozygous variants, i.e., rs2228000 and rs2228001, were found in its protein-coding region. According to the Human Genetic Variation Database, their allelic frequencies in the Japanese population were 23.3% and 60.5%, respectively [21]. It is therefore unlikely that these *XPC* variants are implicated in the XP phenotype. However, whole-exome sequencing analysis identified a compound heterozygous state in the *ERCC2* locus, which has been recognized as the causative gene for XP-D [28]. *ERCC2* is also known as *XPD*. Mutations in the locus cause several hereditary conditions, such as cerebrooculofacioskeletal syndrome, trichothiodystrophy, and xeroderma pigmentosum [29]. In this last condition, mutations have usually been found in either of two Rad51/RecA-like domains, designated as HD1 and HD2, which are responsible for its helicase activity [7]. Both of the mutations described here are situated within HD2. The single-nucleotide mutation, rs758439420, was reported as a homozygous in a German patient with XP-D [30]. In XP40OS, the deleterious SNV along with the 23-bp deletion found on the other allele would impair the enzymatic activity in the Japanese patient. This compound heterozygous state is consistent with the fact that his parents were not consanguineous [14].

An analysis of the mutational signature revealed that XP40OS had accumulated numerous C-to-T or G-to-A transitions. This observation held true in the XP3OS cell line, but those in the dipyrimidine contexts were seen conspicuous exclusively in these XPA-deficient cell lines. Although both the XPA and ERCC2 proteins are responsible for nuclear excision repair, the signature difference seems to reflect each enzyme’s unique molecular function. The XPC DNA repair processes are initiated by recognition of a damage, then the double strand is unwound with the ATP-dependent helicase ERCC3 (XPB) and subsequently with another helicase, ERCC2 (XPD) [31]. Then, XPA assists in expanding the unwound region. Scarcity of dipyrimidine-related mutations compared to the XPA-deficient cells might suggest that the double-strand distortion caused by formation of dipyrimidine makes the helicase activity of ERCC2 dispensable; XPA may be able to recognize and repair dipyrimidine sites without the help of ERCC2. UV exposure causes not only dipyrimidine formation, but also various types of mutations including C-to-T transitions, which was abundant in the XP40OS cell lines.

Searching for retrotransposed genes revealed that the iPSC line of XP40OS had the largest number of candidates; 11 that were uniquely or exclusively found in this line. This observation was the most distinctive feature of the line. XPB-deficient and ERCC2-deficient cell lines exhibited high transduction efficiency and excess accumulation of retroviral cDNAs [23]. In addition, using L1 episomal vectors, XPA, XPC, and ERCC2-deficient cells showed increased numbers of insertions of DNA fragment [32]. It seems likely that ERCC2 functions to defend the genome from the integration of DNA fragments. The ERCC1–ERCC4 heterodimer, which works as endonuclease in downstream of XPA, is also known to reduce retrotransposition of LINE-1 [33]. The mechanism for retrotransposon suppression by ERCC2 is unclear, but it is possible that ERCC2 plays a key role in prevention against insertion of undesirable DNA fragments, making good use of the DNA repair process.

Deletion of *SSL2* and *RAD3*, which are yeast homologs of human *XPB* and *ERCC2*, respectively, gave rise to elevated stability and increased integration of viral cDNAs [34, 35]. *SGS1*, a yeast homolog of human *WRN*, is a DNA helicase responsible for telomere maintenance and was found to repress transcription of retroelements. In other words, it likely inhibits retrotranspositions [36]. Defense mechanisms against retrotranspositions might be shared among the RecQ helicase family including WRN, XPB, and ERCC2. Our analysis suggests that the ERCC2-deficient iPSC line became susceptible to integration of DNA fragments including cDNAs synthesized by endogenous reverse transcriptases. If retrovirus vectors, instead of integration-free Sendai virus, had been employed in the present study, we might have observed a high efficiency of iPSC generation [16]. In the parental XP40OS cell line, however, the number of detected retrotransposed genes detected was comparable to those of the ATM-deficient, XPA-deficient, and control samples. One retrotransposition event certainly occurs in a single cell. With increasing passage number, fibroblast cultures can accumulate a heterogeneous set of retrotransposition events which are mostly present in a low fraction of cells. In contrast, iPSC lines are obtained through cell cloning, and so such somatic events are easier to be detected [8, 15, 18]. It is expected that such somatic events are detected exclusively in iPSCs. Our reprogramming analysis provides a method that can evade laborious single-cell DNA analyses.

Retrotranspositions generally cause more significant changes in phenotype than single-nucleotide or short indel mutations do. Although whole-exome sequencing was enough to detect large-scale mutations involving exon-like sequences, whole-genome sequencing is required to determine genome-wide degree of retrotranspositions of other sequences, such as transposons. In any case, exploiting the clonality of iPSCs and whole-exome sequencing, we have obtained a unique cell line susceptible to integration of DNA fragments, which could be applied to industrial purposes, such as drug development [9, 11]. It is also noteworthy that cellular therapy products may accumulate such mutations during the propagation process in manufacturing.

## Conclusions

Retrotranspositions of protein-coding genes are thought to be present due to the existence of numerous processed pseudogenes in both animals and plants. Unlike retrotransposons including *Alu* and LINE-1, direct evidence of such retrotransposition events has not been reported to date. We herewith report a large number of retrotranspositions of protein-coding genes in cells derived from xeroderma pigmentosum patients. Cell cloning by using the iPSC technology enables us to observe such mutations that have appeared recently in the individual and resulted in quite low allele frequencies in bulk. At least a few retrotranspositions were detected in each control sample. In human somatic cells, retrotranspositions of endogenous genes might be more frequent than expected so far.

## Data Availability

The datasets used and/or analyzed during the current study are available from the corresponding author on reasonable request.

## Abbreviations

iPSC: Induced pluripotent stem cell
XP: Xeroderma pigentosum
UV: Ultraviolet
XP-A: Xeroderma pigmentosum complementation group A
XP-B: Xeroderma pigmentosum complementation group B
XP-C: Xeroderma pigmentosum complementation group C
XP-D: Xeroderma pigmentosum complementation group D
XP-F: Xeroderma pigmentosum complementation group F
XP-V: Xeroderma pigmentosum complementation group V
NER: Nucleotide excision repair
